# Barriers to Modern Contraceptive Use in Kinshasa, DRC

**DOI:** 10.1371/journal.pone.0167560

**Published:** 2016-12-01

**Authors:** Mbadu Muanda, Parfait Gahungu Ndongo, Leah D. Taub, Jane T. Bertrand

**Affiliations:** 1 Programme National de Sante de l’Adolescent (National Program for Adolescent Health), Ministry of Health, Kinshasa, Democratic Republic of Congo; 2 L’Institut Supérieur de Développement Rural, Kongo Central, Democratic Republic of Congo; 3 Department of Global Community Health and Behavioral Sciences, Tulane University School Public Health and Tropical Medicine, New Orleans, Louisiana, United States of America; 4 Department of Global Health Management and Policy, Tulane University School Public Health and Tropical Medicine, New Orleans, Louisiana, United States of America; Karolinska Institutet, SWEDEN

## Abstract

Recent research from Kinshasa, DRC, has shown that only one in five married women uses modern contraception; over one quarter have an unmet need for family planning; and almost 400 health facilities across Kinshasa report that they provide modern contraception. This study addresses the question: with reasonable physical access and relatively high unmet need, why is modern contraceptive prevalence so low? To this end, the research team conducted 6 focus groups of women (non-users of any method, users of traditional methods, and users of modern methods) and 4 of husbands (of users of traditional methods and in non-user unions) in health zones with relatively strong physical access to FP services. Five key barriers emerged from the focus group discussions: fear of side effects (especially sterility), costs of the method, sociocultural norms (especially the dominant position of the male in family decision-making), pressure from family members to avoid modern contraception, and lack of information/misinformation. These findings are very similar to those from 12 other studies of sociocultural barriers to family planning in sub-Saharan Africa. Moreover, they have strong programmatic implications for the training of FP workers to counsel future clients and for the content of behavior change communication interventions.

## Introduction

The DRC is the third most populous country in sub-Saharan Africa, with roughly 77 million people [1]. The country faces high population growth, increasing at a rate of 3.1% per year, which results in a doubling of the population every 22 years. The total fertility rate (TFR) is high at 6.6 children. As of 2013–14 only 7.8% of married women of reproductive age nationwide used modern contraception [2].

Kinshasa, the capital city of the DRC, has an estimated population of approximately 10 million people. The 2013–14 DHS showed Kinshasa to have lower fertility (TFR of 4.4 children) and a higher modern contraceptive prevalence rate (mCPR of 19.0%) than any other province in the country. (In 2015 the provinces were subdivided, so that currently the DRC has 26 provinces. The boundaries of Kinshasa province remained unchanged.) Unmet need was 27.7% among women married or in union aged 15–49 and 42.8% among sexually active unmarried women aged 15–49 in Kinshasa. Although mCPR is similar to other major cities in francophone sub-Saharan Africa, it remains low in comparison to the capital cities of nearby anglophone countries, such as Nairobi, 58.3% [3], Harare, 58.2% [4], Lusaka, 54.7% [5], and Kampala, 40.2% [6].

A 2013 facility-based survey—attempting to identify the “universe” of health facilities providing FP services—yielded 395 sites that reported to offer FP [7]. Subsequent mapping demonstrated gaps in access but showed 1 to 26 FP facilities per health zone (HZ) in all 35 HZ of the city, with a mean of 11.3 health facilities per HZ. Of these 395 facilities, the mean number of methods offered per facility was 3.6. Of the total, 56% carried the implant, a method that is fast growing in popularity. The health system in DRC is made up of three levels: central, intermediate and peripheral. The HZ is equivalent to the peripheral or operational level of the health system. Each HZ includes a general referral hospital and multiple health centers and varies in size by rural or urban locale.

These different statistics led program managers and researchers to ask the perplexing question: With reasonable physical access to modern contraception (at least in the highly populated areas of the city) and high unmet need, why is modern contraceptive prevalence so low in Kinshasa? According to a population-based survey conducted in 2014, the primary reasons for non-use among women of reproductive age were being unmarried (35.7%), infrequent/no sexual activity (27.1%), lack of knowledge (16.4%), and fear of side effects (13.5%) [8]. While useful as a starting point, these data fell short of revealing the attitudes, opinions, beliefs, fears, and cultural norms that explain contraceptive use dynamics in Kinshasa. Thus, the current study was conducted using qualitative methods to better understand the barriers to modern contraceptive use among married women and men in Kinshasa, in neighborhoods where physical access was relatively good.

## Methodology

The study was based on ten focus group discussions among women and husbands of women aged 20–34 who had at least two children. (In addition, eight focus groups were conducted among unmarried females and males aged 15–19; the findings are presented in a separate publication.) The rationale was to obtain data from adults in their peak child-bearing years. The criterion of having at least two children was intended to increase the likelihood that participants would have considered some means of pregnancy prevention.

Having previously studied physical access to contraceptive services [7], for the current research we purposely selected two HZs that were densely populated, centrally located, and had better-than-average access to contraceptive services: Bumbu and Kalamu II. In February 2015 ten focus groups were conducted, based on quota sampling (evenly divided between Bumbu and Kalamu II). The composition of the focus groups was as follows:

- Married women aged 20–34 with at least two children (6 groups) who were:
  - Not using any form of contraception (modern or traditional) (2 groups)
  - Using a traditional method (rhythm or withdrawal) (2 groups)
  - Using modern contraception other than condoms (2 groups)
- Husbands of women aged 20–34 with at least two children who were using a traditional method (rhythm or withdrawal) (2 groups)
- Husbands in non-user unions with women aged 20–34 with at least two children (2 groups)

Each focus group lasted an average of one hour and forty-five minutes, and included 10 participants, for a total of 100: 60 women and 40 men. Given the dominant role of men in DRC society, it is possible but unlikely that their wives would be using contraception without their knowledge.

Participant recruitment for the study varied by the profile of focus group participants. To recruit participants using a modern contraceptive method, the data collection team visited public and private health facilities in each HZ. From client records, the team developed a list of names of women users of FP and their addresses. The team then traveled to each address to invite these women to take part in the group discussion and provide them with the date, time and location for the focus group session. Non-users and husbands in non-user unions were recruited directly from the community; recruitment staff, consisting mainly of nurses, asked women and men about their current use of modern methods, and if they met the criteria (of non-use), invited them to participate in the focus group sessions. Participants were selected from different areas within a given HZ to minimize the chance of knowing each other, and male and female participants were selected from different households.

The data collection team consisted of moderators and note-takers experienced in focus group discussions. Prior to conducting the sessions, they underwent a three-day training that covered focus group methodology, the study protocol and discussion guides, translation of specific questions into the local language, role plays, pre-testing and modifications to the discussion guides.

A moderator of the same sex as the participants led each focus group, while an assistant took notes. The sessions took place at the neighborhood health center in Bumbu and at a community meeting space in Kalamu II. Respondents did not receive remuneration, but were provided snacks and refreshments at the end of the focus group discussion.

The discussion guide explored the following topics: positive and negative attitudes towards use of modern and traditional contraceptive methods; perceptions of method effectiveness; social acceptability of FP use; user preferences; and barriers and obstacles to FP use. All sessions were conducted in the local language and audiotaped. Moderators then transcribed the discussions from the local language to French. Moderators were fluent in both the local language and French, ensuring translation accuracy. Content was organized into a matrix based on themes and sub-themes, allowing for identification of key trends and patterns. Findings were then synthesized and prepared into the descriptive analysis presented below.

Prior to data collection, the study was approved by the human subjects committees of Tulane University (IRB reference number: 14–669918) and of the University of Kinshasa School of Public Health (IRB reference number: ESP/CE/057/2014). During data collection and analysis, study personnel clarified study objectives and ensured confidentiality for participants. Each participant signed a consent form prior to his or her participation in the focus groups.

## Results

### Barriers to modern contraceptive use

The hours of focus group discussions and pages of transcriptions were synthesized to identify the primary reasons for non-use of contraception among married couples in Kinshasa. Although qualitative methods don’t lend themselves to rank-ordering, the frequency and nature of comments by participants pointed to the following five reasons in approximate order of importance: (1) fear of side effects, (2) cost of methods, (3) sociocultural norms, (4) influence of family members against modern method use, and (5) lack of knowledge about the types and source of contraceptive methods and misinformation.

#### Fear of Side Effects

Fear of side effects emerged as the leading reason for nonuse of modern contraception. A few women had previously used a modern method and had experienced side effects (e.g., bleeding from the use of Depo-Provera). Other participants objected to the chemicals in modern contraception and their potential effects on a woman’s health:

I cannot [approve] use of these methods because too many chemicals in the body leads to complications and may also disrupt the period cycle of women, especially after five years of use.

(Husband in non-user union, Kalamu II)

A very deep-seated concern was the potential of contraceptives to cause sterility.

I prefer withdrawal with my husband…We do not use modern methods because we learned that these make women sterile, disrupt the functioning of their body.

(Female/traditional method user, Bumbu)

My friend has used modern contraceptive methods and now she has experienced infertility.

(Female/non-user, Kalamu II)

I have learned that people who use these methods have complications such as cancer, [and] sterility, so I cannot use these methods.

(Female/traditional method user, Bumbu)

I refuse to let my wife use modern methods. Her sister used them, but she is unrecognizable for 6 years. She grew bigger, she can no longer conceive and for these reasons I am not allowing her to use them.

(Husband of traditional method user, Kalamu II)

In addition to sterility, a few participants mentioned that modern contraceptives can cause cancer:

It is my mother who discouraged me. She said that as I only have two children, I must not use these methods. I have to wait for at least 4 to 5 children. Besides, I can’t forget what I’ve heard, that these methods have caused cancer in some people, which is why I refused to use these methods.

(Female/non-user, Bumbu)

#### Cost of methods

A second major barrier to modern contraceptive use was cost; it was cited most frequently in connection with the implant, the preferred method for many potential contraceptive users. The price of methods including the implant varies depending on the service location. As several participants stated:

When we learned that the implant cost $15, I was discouraged and I was no longer interested in these methods because it was too expensive for me.

(*Female/non-user*, *Bumbu*)

I cannot go to the health center because $20 is asked for the implant [which] is the money I need for sales.

(Female/non-user, Bumbu)

For me, if the price is within my ability to pay, I can buy and use them, but if it's too expensive, I wouldn’t know how to [obtain them], because I do not have the means.

(Female/non-user, Bumbu)

People are told that these products are free but when they arrive, they are asked to pay the money and after they are discouraged and they do not come.

(Female/non-user, Bumbu)

Apart from the lack of information is the price. Some products are expensive, it is sometimes beyond $10.

(Husband of traditional method user, Bumbu)

Some women resorted to traditional methods in the face of a related barrier: the requirement for a medical prescription. In pharmacies in Kinshasa, condoms, and, in most cases, the pill, Depo-Provera, implant and IUD are provided to women without a prescription. However, in some cases, pharmacies require women to have a prescription. When asked if she had ever purchased modern methods in a pharmacy, one woman currently using traditional methods recalled:

I [sometimes] buy the pill because I have good relations with the salesperson in the pharmacy, but there are times when [the pharmacy] requires me to have a medical prescription.

(Female/traditional method user, Bumbu)

#### Sociocultural norms

In strongly pronatalist countries, sociocultural norms often influence decision-making regarding family size. In the different focus groups, participants presented their opposition to modern contraceptive use in terms of African culture. As several male participants explained, the man has the predominant role in family decision-making, including childbearing, because in African culture, he is the head of the family. Few husbands believed that the decision over the number of children should come from the couple.

It is the man who decides the number of children because it is he who works, not the mother because she…lives from her husband’s earnings.

(Husband in non-user union, Bumbu)

For me, the decision [to use family planning] comes from the man, and all decisions revert to him because he is the head of the family, the head of the household…If he says, ‘I need to stop here for lack of financial means,’ it’s for him to decide.

(Husband in non-user union, Bumbu)

I am the man, I am the master of the family, it is I who manages everyone. The woman should not make decisions for me.

(Husband in non-user union, Kalamu II)

There are women who speak of these methods to their husbands. If the man refuses, the woman must bow, but if the man accepts, the woman can use…

(Husband of traditional method user, Kalamu II)

As is outlined above, it is common in the DRC that the husband is dominant in decision-making, especially with regard to number of children and by extension, family planning. As one woman explains:

There are women who do not go to the health center because their husbands have forbidden them, [and] they are afraid to disobey.

(Female/modern method user, Bumbu)

Several husbands also cited the need to respect the natural order established by God and the woman’s natural cycle; to use modern methods in African culture would be disrespectful.

Me, I consider our African culture. We grew up with our mothers not using modern methods, but they knew to space births, they were smart. We also must find out a way to prevent pregnancies one after the other.

(Husband of traditional method user, Bumbu)

We are Africans, it is the man who decides in the home…[but] let’s not forget in terms of children it is God who gives…

(Husband in non-user union, Kalamu II)

However, some husbands did advocate joint decision-making with regard to number of children.

It is the man and the woman who must agree to avoid occurrence of a pregnancy one way or another by choosing a method of birth spacing.

(Husband in non-user union, Bumbu)

It is the woman who must decide on the use of a contraceptive method. It is she who must propose the idea of [birth] spacing to the man.

(Husband in non-user union, Kalamu II)

Whereas many men considered it their prerogative to decide the number of children, they felt it was the woman’s responsibility to deal with the mechanics of family planning:

Me, I never went to take a contraceptive method in these health centers because it is a matter of women.

(Husband in non-user union, Bumbu)

#### Pressure from family to avoid modern contraception

A number of participants also cited the negative opinions of others–husbands, sisters, mothers, and others in the community–as having influenced their own non-use of modern contraception. Women are pressured by others to avoid family planning because of the desire for large families and the perceived dangers of modern contraceptive use:

My older brothers forbade me from using [modern methods] because they know of women who used these methods and have become sterile. There has now been 10 years that these women cannot conceive.

(Husband in non-user union, Kalamu II)

Since my big sister had complications, she advised me not to take these methods.

(Female/non-user, Bumbu)

My husband told me: if you get an injection, you will become unfaithful, you will start dating other men because you know that you will not fall pregnant.

(Female/non-user, Kalamu II)

#### Lack of knowledge and misinformation

Lack of information and misinformation are additional barriers to contraceptive use. This category includes lack of knowledge about the different types and sources of contraception, and misinformation that certain medications prevent pregnancy.

A few participants–both female and male–had not heard of a modern contraception or they lacked detailed knowledge about it:

I have never used these modern methods because I did not have the information…but as I now have the information I will use.

(Female/non-user, Bumbu)

Me, I have not used these [modern] methods in the past because I did not know them, only now I learn that these methods exist and can help prevent children.

(Husband in non-user union, Bumbu)

Similarly, several non-users and husbands in non-user unions had no knowledge of health centers offering modern methods in their community. By contrast, other male and female participants knew about modern contraceptives and locations that offered them, but had not visited them and instead relied on traditional methods.

False information about products believed to be contraceptives (but are not) has also led to disillusionment over the effectiveness of modern methods. For example, several participants mentioned that Decaris, tetracycline and tanzol were contraceptives. Decaris and tanzol are pharmaceutical products used for deworming, but some women believe them to be effective in killing sperm (and thus preventing pregnancy). A number of women and husbands of women relying on traditional methods had purchased products from a pharmacy to prevent pregnancy in the past and used them, only to have the woman became pregnant. In their minds, this was proof that modern contraception is ineffective. As one participant explained:

Me, I already bought Decaris, tetracycline, and even the pill in a pharmacy and the last time unfortunately I had my child, who is now 2 years.

(Female/non-user, Bumbu)

### Women who had overcome barriers and were using modern contraception

The study design included two focus groups with women currently using a modern contraceptive method, to glean insight into how they might have overcome similar barriers to become modern contraceptive users.

Communication with and support from the husband for contraceptive use emerged as a key factor among these women. Even in the face of opposition from other family members, friends and community members, if the husband was supportive of a woman’s desire to use modern contraception, the arguments of others had little influence.

The people in the neighborhood told me not to use these methods because they lead to infertility, but…as I had already obtained the consent of my husband…I went to take the method.

(Female/modern method user, Bumbu)

I discussed with my husband and he agreed. It is he himself who gave me money to go to the health center to take my method. I took the implant because we wanted to rest as long as possible [space births] in order to properly raise our children.

(Female/modern method user, Bumbu)

Husbands were reportedly favorable toward the wife’s use of family planning for several reasons: perceived importance of birth spacing for the woman’s wellbeing, difficult economic conditions, and desire to limit childbearing due to high number of children already in the family.

However, some women were using contraception without informing their husbands or despite the husband’s opposition. As one woman user stated:

To be honest, I had not informed my husband, I did it on my own. As I know well, if I had told him, he would necessarily be opposed…

(Female/modern method user, Kalamu II)

Users cited other factors that influenced their decision to use a modern contraception: perceived advantages of modern methods (i.e., protection from pregnancy) and proximity of sources of contraceptives (e.g., health centers and pharmacies) to the women’s place of residence, which for many users outweighed perceived disadvantages of side effects (i.e., bleeding and cessation of menstruation). The manner in which the woman was received at the source of supply also played a positive role. As one woman user shared:

I was scared before going for my method at the health center, but upon check-in the health staff received me well. I was also reassured by the effectiveness of methods.

(Female/modern method user)

Despite these enabling factors, price remained a barrier for some users to access their preferred (long-term) method, which led them to settle for another method. As one user explained:

When I came to the health center, health staff introduced me to all the methods and the price for each. I wanted the implant, but as I did not have much money, I chose Depo-Provera.

(Female/modern method user, Bumbu)

## Discussion

The findings from this study provide valuable insights into the reasons that four in five married women in Kinshasa do not use modern contraception, despite the fact that close to one-third of married women have an unmet need for family planning. From this analysis the key barriers are fear of side effects (especially sterility), cost of the method, cultural norms favoring large families, pressure from family members to avoid modern contraception, and lack of knowledge about the type and source of contraceptive methods, as well as misinformation.

A review of the peer-reviewed literature produced 12 qualitative studies published to date on barriers to family planning use in sub-Saharan African countries; the countries represented were Ethiopia, Ghana, Malawi, Nigeria, Rwanda, Uganda, and Burkina Faso. To illustrate the similarity in findings between the current Kinshasa study and previously published research, we created a table that lists the 12 articles by author and country (see Table 1). We then listed in the column headings the five barriers found in the Kinshasa study: fear of side effects, cost of the method, cultural norms (especially the role of the husband in decision-making), pressure from family members, and lack of knowledge or misinformation. We also allowed for additional factors to be added in the sixth and seventh columns (quality of services and ambivalence). Table 1 shows strong similarities across countries in the barriers to contraceptive use. Specifically, on five of the seven factors (all except cost of method and quality of services), at least half of the 12 studies reported the same barrier. Fear of side effects, cultural norms and pressure from family members were the three most frequently cited (in nine of the 12 studies). Of note, fear of side effects included both experienced and perceived. By contrast, only four of the 12 cited the cost of methods. A study conducted in three HZs in rural areas of DRC far from the capital city also identified four of the five same factors (all except cost) [9]. The consistency of findings across sub-Saharan African countries further validates the findings from the Kinshasa study. Moreover, it identifies for program managers and behavior change specialists the areas to address in future demand creation activities.

**Table 1:** Findings from qualitative studies on barriers to family planning use in sub-Saharan Africa.

Author(s)	Country	Type of barrier						
		Cultural norms	Costs associated with FP services	Fear of side effects	Pressure from family members	Lack of knowledge or misinformation	Quality of services^a^	Ambivalence
Gebremariam and Addissie, 2014 [10].	Ethiopia			X		X	X	X
Adongo et al, 2014 [11].	Ghana	X		X	X	X		X
Adongo et al, 2014 [12].	Ghana	X		X	X	X		X
Hennick and Madise, 2005 [13].	Malawi	X	X	X	X		X	
John, Babalola, and Chipeta, 2015 [14].	Malawi	X		X	X			X
Aransiola, Akinyemi, and Fatusi, 2014, [15].	Nigeria	X			X			X
Okwor and Olaseha, 2009 [16].	Nigeria	X		X	X			
Diamond-Smith, Campbell, and Madan, 2012 [17].	Nigeria, Nepal and India			X	X	X		
Farmer et al, 2015 [18].	Rwanda	X	X	X	X	X	X	X
Kabagenyi et al, 2014 [19].	Uganda	X		X	X	X	X	X
Pitorak, Lubaale, and Gurman, 2014 [20].	Uganda	X	X					X
Rossier, Senderowicz, and Soura, 2014 [21]	Burkina Faso	X	X	X	X	X	X	X

^a^“Quality of services” as a barrier includes limited method choice, stock-outs, long wait times, and poor reception and lack of quality counseling by health staff.

In comparing the qualitative findings from this study with the quantitative findings from the PMA2020 survey (a population-based survey conducted in Kinshasa at roughly the same time), two factors appeared in both: fear of side effects and lack of knowledge. The qualitative study further identified cost of the method, sociocultural norms (relating to male decision-making and religious beliefs), and pressure from family members not to use modern contraception.

Qualitative research has numerous limitations that are well documented elsewhere. It cannot be generalizable to other populations [22–24]. The small number of persons participating in the research increases the possibility of selecting atypical participants. On sensitive topics participants may be reticent or feel embarrassed to reveal their true feelings. One or two members of the focus group may dominate the conversation if the moderator is not skilled in controlling or countering this situation [22–24]. Nonetheless, the findings from this study underscore the value of qualitative research in understanding the nuances of barriers to contraceptive use.

**Fig 1:**
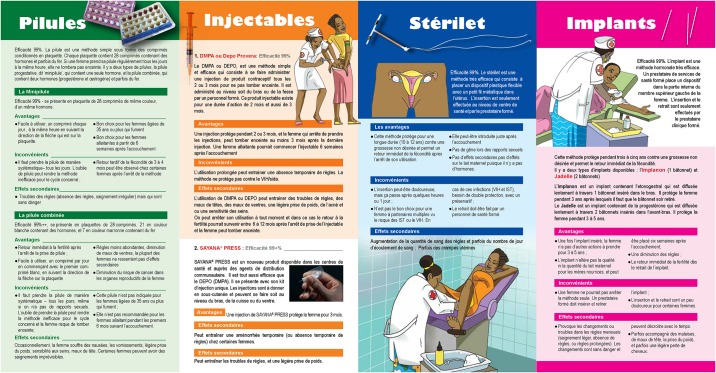
Leaflets for low-literacy audiences on widely used methods.

The study has several programmatic implications, some of which are already being implemented or are in the planning stages. To address both lack of knowledge about the methods and fear of side effects, a series of leaflets has been produced for low-literacy audiences that describes the range of the most widely used methods (Fig 1) as well as brochures for four specific methods. Regarding the cost barrier, several of the programs offering contraceptive methods in sites throughout the city are exploring ways to provide contraceptives free of charge, either routinely or in period “campaign days.” Ideally, it will be possible to segment the market using the total market approach [25–26], such that those able to pay for contraception will do so (paying full or subsidized prices), whereas those unable to pay will have access to a source of free contraception.

Cultural norms that support large families may be the most difficult of barriers to address. In particular, the belief that modern contraception retards or jeopardizes future fertility deserves particular attention in future counseling and communication campaigns, as it threatens the deeply rooted value of procreation. In contrast to other societies in which couples who attain their desired number of children are willing to adopt a permanent method (i.e., female or male sterilization), the potential for future childbearing remains all-important in a society where the future is unpredictable. With high levels of infant and child mortality, a lack of resources to reply to acute crises, widespread food shortages, and no publically financed safety net, families remain unsure of what the future will hold. Moreover, the DRC has a government that lacks the means to support human and food security at the most basic levels and a justice system that does not have the resources to enforce laws and regulations, with corruption pervasive in public and private sector institutions.

Despite strong pronatalist norms, the experience from other countries within sub-Saharan Africa and other regions of the world illustrates the steady evolution toward smaller families, especially as couples embrace the quantity-quality trade-off (of having fewer children to be able to give them a better quality life) [27]. Urbanization also contributes to reducing family size: even within the DRC, the TFR is considerably lower for Kinshasa (4.4 children) than the national TFR (6.6 children). Despite the deep-rooted value placed on large families in much of sub-Saharan Africa, many countries have begun the gradual advance toward increased modern contraceptive prevalence. Barriers to contraceptive use are real, and they deter many women and men from using family planning. Yet the gradual increase in modern contraceptive use, matched by the steady decrease in fertility rates in countries worldwide, is evidence that these barriers are not insurmountable.

## References

[1] United Nations. (2015). World population prospects: the 2015 revision, key findings and advance tables. New York: United Nations; 2015. 59 p. Report No.: ESA/P/WP.241

[2] Ministère du Plan et Suivi de la Mise en œuvre de la Révolution de la Modernité (MPSMRM), Ministère de la Santé Publique (MSP) et ICF International (2014) Enquête Démographique et de Santé en République Démocratique du Congo 2013–2014. Rockville, Maryland: MPSMRM, MSP et ICF International http://dhsprogram.com/pubs/pdf/FR300/FR300.pdf

[3] Kenya National Bureau of Statistics (KNBS) and ICF Macro (2015) Kenya Demographic and Health Survey Key Indicators 2014. Calverton, Maryland: KNBS and ICF Macro https://dhsprogram.com/pubs/pdf/PR55/PR55.pdf

[4] Zimbabwe National Statistics Agency (ZIMSTAT) and ICF International (2012) Zimbabwe Demographic and Health Survey 2010–11. Calverton, Maryland: ZIMSTAT and ICF International Inc http://dhsprogram.com/pubs/pdf/FR254/FR254.pdf

[5] Central Statistical Office (CSO) [Zambia], Ministry of Health (MOH) [Zambia], and ICF International (2014) Zambia Demographic and Health Survey 2013–14. Rockville, Maryland: Central Statistical Office, Ministry of Health, and ICF International. https://www.dhsprogram.com/pubs/pdf/FR304/FR304.pdf

[6] Uganda Bureau of Statistics, ICF International (2012) Uganda Demographic and Health Survey 2011. Calverton, Maryland, USA: UBOS and ICF International https://dhsprogram.com/pubs/pdf/FR264/FR264.pdf

[7] Kayembe P, Babazadeh S, Dikamba N, Akilimali P, Hernandez J, Binanga A, et al Family planning supply environment in Kinshasa, DRC: survey findings and their value in advancing family planning programming. Glob Health Sci Pract. 2015 12; 3(4):630–645. 10.9745/GHSP-D-15-00298 26681709 PMC4682587

[8] PMA2015/Kinshasa-round 3: key family planning indicator brief. Baltimore: Bill & Melinda Gates Institute for Population and Reproductive Health, Johns Hopkins Bloomberg School of Public Health; 2015.

[9] Bertrand J, Muanda M, Babasadeh S, Yongho AM. Analyzing supply and demand for family planning in ASSP–supported health zones in the DRC: a case study. Tulane University; 2016.

[10] Gebremariam A, Addissie A. Knowledge and perception on long acting and permanent contraceptive methods in Adigrat Town, Tigray, Northern Ethiopia: a qualitative study. Int J Family Med. 2014; 2014:1–6. 10.1155/2014/878639 PMC4130128 25140252

[11] Adongo PB, Tabong PT, Azongo TB, Phillips JF, Sheff MC, Stone AE, et al A. Front Public Health. 2014 9; 2:1–7. 25250307 10.3389/fpubh.2014.00137 PMC4155786

[12] Adongo PB, Tapsoba P, Phillips JF, Tabong PT, Stone A, Kuffour E, et al "If you do vasectomy and come back here weak, I will divorce you": a qualitative study of community perceptions about vasectomy in Southern Ghana. BMC Int Health Hum Rights. 2014 5; 14:1–8. 10.1186/1472-698X-14-16 PMC4019590 24885663

[13] Hennink M, Madise N. Influence of user fees on contraceptive use in Malawi. African Population Studies. 2005; 20(2):101–123.

[14] John NA, Babalola S, Chipeta E. Sexual pleasure, partner dynamics and contraceptive use in Malawi. Int Perspect Sex Reprod Health. 2015 6; 41(2):99–107. 10.1363/4109915 26308262

[15] Aransiola JO, Akinyemi AI, Fatusi AO. Women's perceptions and reflections of male partners and couple dynamics in family planning adoption in selected urban slums in Nigeria: a qualitative exploration. BMC Public Health. 2014 8; 14:1–14. 25148699 10.1186/1471-2458-14-869 PMC4165936

[16] Okwor EU, Olaseha IO. Married men's perception about spousal use of modern contraceptives: a qualitative study in Ibadan Northwest local government area, Southwest Nigeria. Int Q Community Health Educ. 2010; 30(3):223–238. 10.2190/IQ.30.3.d 20860981

[17] Diamond-Smith N, Campbell M, Madan S. Misinformation and fear of side-effects of family planning. Cult Health Sex. 2012; 14(4):421–433. 10.1080/13691058.2012.664659 22390371

[18] Farmer DB, Berman L, Ryan G, Habumugisha L, Basinga P, Nutt C, et al Motivations and constraints to family planning: a qualitative study in Rwanda's Southern Kayonza district. Glob Health Sci Pract. 2015 5; 3(2):242–254. 10.9745/GHSP-D-14-00198 26085021 PMC4476862

[19] Kabagenyi A, Jennings L, Reid A, Nalwadda G, Ntozi J, Atuyambe L. Barriers to male involvement in contraceptive uptake and reproductive health services: a qualitative study of men and women's perceptions in two rural districts in Uganda. Reprod Health. 2014 3; 11(1):1–9. 24597502 10.1186/1742-4755-11-21 PMC3946591

[20] Pitorak H, Lubaale SK, Gurman TA. "It depends on your pocket:" findings from a qualitative study in Uganda exploring women's and health care providers' perspectives on family planning. Health Care Women Int. 2014; 35(3):234–248. 10.1080/07399332.2012.736575 23530463

[21] Rossier C, Senderowicz L, Soura A. Conflicted fertility preferences and contraceptive use among Burkina Faso’s urban poor. Paper presented at the Population Association of America Annual meeting; Boston, Massachusetts; 2014 May 1–3.

[22] Patton MQ. Qualitative evaluation and research methods. Thousand Oaks: Sage Publications, Inc; 2002.

[23] Mack N, Woodsong C, MacQueen KM, Guest G, Namey E. Qualitative research methods: a data collectors field guide Research Triangle Park: Family Health International; 2005.

[24] Morgan DL. Focus groups as qualitative research. Thousand Oaks: Sage Publications, Inc.; 1997.

[25] Barnes J, Vail J, Crosby D. Total market initiatives for reproductive health. Bethesda: Abt Associates; 2012.

[26] Chapman S, Jafa K, Longfield K, Vielot N, Buszin J, Ngamkitpaiboon L, et al Condom social marketing in sub-Saharan Africa and the total market approach. Sex Health. 2012 3; 9(1):44–50. 10.1071/SH10165 22348632

[27] Becker GS. An economic analysis of fertility In: Universities-National Bureau, editor. An economic analysis of fertility. Princeton: Princeton University Press Princeton; 1960 p. 209–240

